# A Java-based tool for the design of classification microarrays

**DOI:** 10.1186/1471-2105-9-328

**Published:** 2008-08-04

**Authors:** Da Meng, Shira L Broschat, Douglas R Call

**Affiliations:** 1 School of Electrical Engineering and Computer Science, Washington State University, Pullman, USA; 2 Department of Veterinary Microbiology and Pathology, Washington State University, Pullman, USA; 3 Center for Integrated Biotechnology, Washington State University, Pullman, USA

## Abstract

**Background:**

Classification microarrays are used for purposes such as identifying strains of bacteria and determining genetic relationships to understand the epidemiology of an infectious disease. For these cases, mixed microarrays, which are composed of DNA from more than one organism, are more effective than conventional microarrays composed of DNA from a single organism. Selection of probes is a key factor in designing successful mixed microarrays because redundant sequences are inefficient and limited representation of diversity can restrict application of the microarray. We have developed a Java-based software tool, called PLASMID, for use in selecting the minimum set of probe sequences needed to classify different groups of plasmids or bacteria.

**Results:**

The software program was successfully applied to several different sets of data. The utility of PLASMID was illustrated using existing mixed-plasmid microarray data as well as data from a virtual mixed-genome microarray constructed from different strains of *Streptococcus*. Moreover, use of data from expression microarray experiments demonstrated the generality of PLASMID.

**Conclusion:**

In this paper we describe a new software tool for selecting a set of probes for a classification microarray. While the tool was developed for the design of mixed microarrays–and mixed-plasmid microarrays in particular–it can also be used to design expression arrays. The user can choose from several clustering methods (including hierarchical, non-hierarchical, and a model-based genetic algorithm), several probe ranking methods, and several different display methods. A novel approach is used for probe redundancy reduction, and probe selection is accomplished via stepwise discriminant analysis. Data can be entered in different formats (including Excel and comma-delimited text), and dendrogram, heat map, and scatter plot images can be saved in several different formats (including jpeg and tiff). Weights generated using stepwise discriminant analysis can be stored for analysis of subsequent experimental data. Additionally, PLASMID can be used to construct virtual microarrays with genomes from public databases, which can then be used to identify an optimal set of probes.

## Background

The majority of DNA microarrays in use today are created from single genomes that do not reflect the genetic diversity of a group of heterogeneous entities. Mixed-DNA microarrays offer an alternative for "capturing" genetic diversity and can be used for classification purposes such as identifying pathogens or determining genetic relationships for epidemiology studies [1-4]. DNA from one or more reference strains or plasmids is shotgun-cloned, and a mixed-genome or mixed-plasmid microarray is generated from randomly selected, PCR-amplified clone inserts [2,3]. Unlike most fingerprinting tools, the mixed-array format permits identification of informative probes that can be retrieved from the clone library for sequencing [5]. However, redundant sequences and limited representation of diversity can limit the application of these tools [3,4]. Fortunately, a growing public database of genomes offers a new opportunity to incorporate non-redundant and diverse sequences into a mixed-microarray format. These arrays can be used to quickly assess the distribution of genetic diversity across multiple species and niches.

This work focuses on the optimal design of classification arrays. By optimal we mean minimizing the complexity and cost of an array by using as few probes as possible while still rendering sufficient information to discriminate between strains or groups of organisms and to avoid bias; the goal is to remove irrelevant probes (probes that contain no useful information) and reduce the number of redundant probes (probes that contain the same information) in such a way that the chosen probes will allow us to perform the desired classification task accurately. Selection of an optimal set of probes is a key factor in designing a successful mixed microarray to suit a particular need. The effects of probe length and the number of probes per gene have been discussed in [6]. A method for finding unique and valid oligonucleotides or probes was proposed in [7], which tries to identify probes for a gene such that there is no similar occurrence in other locations of a genome. A tool for choosing optimal DNA oligos is reported in [8], which identifies oligo sequences that occur in members of the target group but not in the non-target group. However, these methods are used for genome-wide probe selection and are not intended to identify minimum probe sets for classification problems.

A number of methods have been introduced for designing optimal probe sets. Pre-filtering methods [9] use clustering of all probes to find similar probe groups. Similar probes are discarded; the remaining probes are ranked, and top-ranked probes are kept for further analysis. A similar method [10] uses K-means to cluster all genes, and the means of different gene clusters are used as prototype genes. The limitation of these methods is that the number of clusters must be specified. A hybrid approach [11] ranks the probes first and selects a set of top-ranked probes. Hierarchical clustering is then used on these probes to generate a dendrogram. The optimal probes are selected by collapsing dense clusters. In this manner a small set of probes is identified that has a similar prediction accuracy to one that uses more probes.

The methods described above identify optimal probes using training data when the structure of the data is given. Such information, however, is usually unavailable for microarray data sets. A tool is still needed to help design mixed microarrays when prior knowledge of a microarray data set is unavailable. The focus of this paper is a software program, PLASMID, used for selecting an optimal set of probe sequences without a *priori *knowledge that will enable correct classification of groups of plasmids or bacteria. Data used to identify probe candidates can be either existing microarray data (or similar hybridization data) or sequence data from a public database such as GenBank. The latter are converted to "probe" sequences, and virtual hybridization is used to generate data for probe selection [1]. To demonstrate the generality of PLASMID, we include an example whereby the program can also be applied to develop a minimum probe set to distinguish between two classes of leukemia using data from an expression array.

## Methods

### Finding meaningful clusters in hybridization data

Finding meaningful clusters of samples (e.g., plasmids) from a given set of hybridization or sequence data is the starting point for the design of an optimal microarray; our tool provides several clustering options. Clustering methods can be divided into two general groups: distance-based methods and model-based methods. Distance-based methods are either non-hierarchical or hierarchical, and each method has its particular strengths and weaknesses. Currently our tool includes the K-means non-hierarchical clustering algorithm and hierarchical clustering by means of Unweighted Pair Group Method with Arithmetic mean (UPGMA), neighbor joining, or Ward's minimum variance method, all of which are widely used in microarray data analysis [12]. Two distance metrics have been implemented, Euclidean distance and Pearson's correlation coefficient, from which users can choose. The distance-based methods listed above are standard clustering techniques. In addition to these, we have also implemented the model-based clustering method described below.

#### Model-based genetic clustering

Distance-based methods are simple to use, and the clustering results are easy to explain. However, it is hard to obtain information about the number of clusters, the confidence level of the clustering results, and so on, from these methods. To avoid some of these issues, model-based clustering methods can be used as an alternative. Model-based clustering methods assume that the data can be clustered according to a set of underlying distributions. These underlying distributions can be modeled, and finding a suitable model can be construed as an optimization problem. We assume that *M *is the underlying model for a data set represented by a matrix X where each row of the matrix represents the data for a given sample (e.g., plasmid). The best clustering result is represented by partition *P *of X. A measure is used to determine which *P *is most likely for X. In our tool the measure is the likelihood of all possible partitions *P*. A number of different optimization methods can be used to find the solution for *P*. In our tool, we have chosen to use a genetic algorithm because of its simplicity and efficiency in addition to its ability to find the optimal solution. Usually model-based clustering methods are based on the Expectation-Maximization (EM) method. However, EM algorithms tend to break down for microarray data because an inversion of the covariance matrix must be performed. In genetic algorithms, a search method is used to circumvent the need for this computation, thereby making genetic model-based methods more stable.

To find the best partition *P *we want to maximize the posterior probability *f*(*P*|X). According to Bayes' theorem, $f(P|\text{X})=\frac{f(\text{X}|P)f(P)}{f(\text{X})}$ where *f*(*P*) is the prior probability. Recasting Bayes' theorem in terms of the likelihood ℒ(X|*P*) gives *f*(*P*|X) ∝ ℒ(X|*P*) *f *(*P*)–that is, the posterior probability is proportional to the product of the likelihood and prior probability. Now if we assume a uniform distribution for *P*, then *f*(*P*) is constant and maximizing the posterior probability *f*(*P*|X) is equivalent to maximizing the likelihood ℒ(X|*P*).

If we assume the rows of the matrix X in each cluster of the partition are independent and identically distributed, we can compute the likelihood of a partition. For this work, we assume the rows in each cluster are normally distributed with mean *μ*_*i *_and variance $\sigma_{i}^{2}$, and we assume a normal distribution for all *μ*_*i *_and an inverse-Γ distribution for all $\sigma_{i}^{2}$. This leads to:

(1) $$ℒ(\text{X}|P)=\prod_{k}\prod_{j}\frac{2\sigma_{0}^{2}}{\Gamma (1)}\frac{{\left(2\pi \right)}^{-(n_{k}/2)}}{\sqrt{n_{k}+1}}\frac{\Gamma (n_{k}/2+1)}{{\left(2\sigma_{0}^{2}+0.5(\sum_{i}x_{k_{i}j}^{2}+\mu_{0}^{2}-\frac{\sum_{i}{\left(x_{k_{i}j}+\mu_{0}\right)}^{2}}{n_{k}+1})\right)}^{\left(n_{k}/2+1\right)}}$$

where *k *is the index of clusters, *j *is the index of probes, *n*_*k *_is the number of samples in the *k*th cluster, *k*_*i *_is the index of samples in the *k*th cluster, and *μ*_0 _and $\sigma_{0}^{2}$ are the overall mean and variance of all the data [13].

Using this as a measure, the genetic algorithm is used to find the partition that maximizes the likelihood. The steps of the genetic algorithm are summarized as follows:

1. Generate *N *random partitions. Each partition is represented by a vector [1 2 1 ⋯] where each term is the index of a cluster.

2. Prior knowledge of pairs of samples highly unlikely to be in the same cluster can be incorporated into the partition likelihood by creating a text file with each pair of samples, together with a small weighting factor, on one line. The weighting factor must be smaller than 1, but how much smaller has to be determined empirically based on the end result. A weighting factor of zero indicates that the pair cannot be in the same cluster.

3. Compute the likelihood ℒ for all partitions.

4. Repeat the following steps until the the maximum iterations (*Max*) has been reached or the difference between the likelihood of two successive iterations is less than *ε*, where *Max *and *ε *are given.

(a) Select the two partitions with the highest scores.

(b) Do crossover and mutation on these two partitions to generate new partitions. Crossover is accomplished by randomly selecting sections of equal length from each partition and exchanging them. Mutation is performed following crossover and is accomplished by randomly selecting one term in each of the partitions and changing it to a different value.

(c) Compute the likelihood ℒ for these two new partitions (offspring).

(d) Replace the two lowest-ranked partitions with the offspring.

Other measures can be used including Bayesian Information Criteria and minimum description length. These measures will be included in future versions of PLASMID.

### Probe ranking for classification

In a DNA microarray data set there are usually many more probes than the number of samples (e.g., plasmids) to be classified, and often some probes either convey no useful information or convey the same information. Thus, in the design of an optimal probe set for sample classification, one objective is to identify and remove irrelevant and redundant probes. In this section, we describe our method for removing irrelevant probes; in the next section redundancy reduction is described.

Irrelevant probes are removed using probe ranking on the clusters of samples obtained in the previous step. There are two basic approaches to probe ranking: filter techniques and wrapper techniques. Because of their simplicity, filter procedures are used most commonly for DNA microarrays. The filter procedure ranks each probe using a metric based on its classification relevance. Top-ranked probes are then selected to perform classification. Numerous filter metrics are described in the literature [14]: probabilistic and distance metrics, dependence measures, scores based on information theory, etc. In our tool, filter metrics are determined using two different statistical tests, the ANOVA-*F *and Brown-Forsythe tests. Other tests considered were the Welch, adjusted Welch, Cochran, and Kruskal-Wallis test statistics [15].

The test statistic is used as a metric to evaluate the discriminating power of a probe. Higher values represent more discriminating probes. For some applications, clusters may include an insufficient number of samples for meaningful statistical analysis. Such cases can be handled by generating random samples that differ only slightly from the original samples. These samples can be included in the statistical analysis and then discarded without compromising the probe ranking procedure. The purpose of adding these samples is for computational convenience only; they do not add more information.

The end result of the probe ranking function is a list of all probes ranked by their classification relevance. At this point, the user can either stop and use some chosen number of the top-ranked probes for the array probe set or continue with probe reduction and stepwise discriminant analysis to remove redundant probes and assign weights to the probes.

### Stepwise discriminant analysis

Probe ranking is used to remove irrelevant probes that convey little or no information. Nevertheless, while the top-ranked probes are informative, at least some of them are likely to convey redundant information. The next task is to remove this unnecessary redundancy. K-means clustering is usually used to cluster samples (e.g., plasmids) as described in an earlier section, but here we use it in a novel way to cluster probes. A set of top-ranked probes is clustered into *κ *groups where the value of *κ *is evaluated empirically to maximize classification accuracy; probes in the same group are highly correlated with each other but uncorrelated or loosely correlated with probes in other groups. The probe closest to the center of a group is chosen to be representative of that group, and the *κ *representative probes are used with stepwise discriminant analysis (SDA) [16] which identifies the optimal probe set G from the *κ *probes. At each step of the SDA, an F statistic is computed for each probe; this value is used to determine whether including the probe or excluding the probe from G will significantly improve sample differentiation. The SDA process starts with an empty probe set G, and an iterative process of adding a probe to G or removing a probe from G continues until no probes can be added or removed. $F_{remove}$ is used for the probes in G, and $F_{enter}$ is used for the probes not in G. The probe in G with the smallest value of $F_{remove}$ less than a chosen threshold value, usually 1.0, is removed; the probe not in G with the largest value of $F_{enter}$ greater than the threshold value is added to G. The formulas used to compute F are:

F values:

(2) $$F_{remove}=\frac{n-r-q+1}{q-1}\frac{\Lambda (G\backslash p)-\Lambda (G)}{\Lambda (G)}$$

(3) $$F_{enter}=\frac{n-r-q}{q-1}\frac{\Lambda (G)-\Lambda (G|p)}{\Lambda (G|p)}$$

Wilks' Λ:

(4) $$\Lambda (G)=\frac{\det(W)}{\det(T)}$$

Within-group covariance matrix:

(5) $$W(G)=\sum_{m=1}^{q}\sum_{m=1}^{n_{m}}\left(x_{mki}-x_{mi.})(x_{mkj}-x_{mj.}\right)$$

Among-group covariance matrix:

(6) $$T(G)=\sum_{m=1}^{q}\sum_{m=1}^{n_{m}}\left(x_{mki}-x_{i..})(x_{mkj}-x_{j..}\right)$$

where *q *is the number of clusters, *n*_*m *_is the number of samples in the cluster *m*, *x*_*mki *_is the value of the *i*th probe for the *k*th sample in the *m*th cluster, *n *is the total number of samples, *r *is the number of probes currently included in G, G|*p *denotes a new group of probes which is obtained by adding the probe *p *to G, and G\*p *denotes a new group of probes which is obtained by removing the probe *p *from G.

At the conclusion of SDA, the optimal probe set is determined based on the prediction accuracy of the selected probes. Because there are typically a small number of samples associated with microarray data, prediction accuracy is computed using the leave-one-out (LOO) cross validation method [11,15]. The set of probes associated with the highest LOO predication accuracy is written to a file together with its associated weights. It is important to note that when SDA is used to obtain the final probe set, the weights associated with the probes must be used for classification of new empirical data obtained using the probes. The probes should not be treated with equal weight.

### Probe selection for a classification microarray

In summary, the steps in our design of an optimal probe set are:

1. Cluster the samples (e.g., plasmids) using microarray or sequence data and select clusters of interest using a hierarchical, non-hierarchical, and/or model-based method. *A priori *clustering is also permitted.

2. Use the probe ranking procedure with the sample clusters to rank the probes for relevance.

3. Repeat K-means clustering of probes for probe reduction until satisfied:

(a) Select *j *top-ranked probes.

(b) Repeat for *κ *in a chosen range:

i. Cluster the *j *top-ranked probes into *κ *clusters.

ii. Choose *κ *representative probes, one from each cluster.

iii. Use SDA to find a set of probes from the *κ *representative probes and compute the LOO prediction accuracy.

4. Save the set of probes associated with the highest LOO prediction accuracy together with its weights. After constructing the optimized microarray, a set of independent control samples should be hybridized to empirically assess the accuracy of the microarray results.

**Figure 1:**
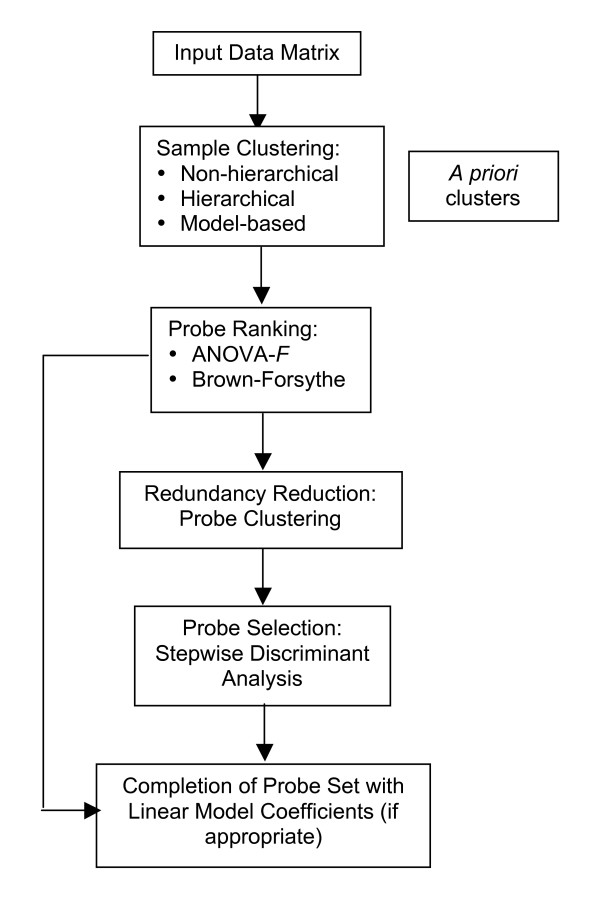
**Flowchart of PLASMID**. Flowchart of the probe selection process using PLASMID.

A flowchart of the process is shown in Fig. 1. It should be pointed out that the optimal number of probes computed by this process does not take into account the effects of noise and other random experimental effects. The sample-to-feature (SFR) ratio gives the minimum number of probes that should be used to create a microarray. The rule of thumb is given by [17]:

$$SFR=\frac{number\;of\;samples}{number\;of\;features}\leq \frac{1}{5}.$$

In this paper we refer to features as probes. The SFR should be used in conjunction with the results to choose the optimal probe set.

## System Overview and Implementation

Our software tool PLASMID is implemented as a Java application. The NetBeans platform was chosen for development because addition of new functions is easily implemented. Also, many of the tasks common to desktop applications are provided by NetBeans. These include user interface management (e.g., menus and toolbars), user settings management, storage management (saving and loading any kind of data), window management, and wizard framework (supporting step-by-step dialogs). Each function is implemented as a NetBeans module and can be installed or removed easily without affecting existing functions. Java is a platform-independent programming language, so although PLASMID has been developed using the Windows operating system, it will be relatively easy to adapt it to other operating systems. We intend to extend PLASMID to both the Linux and Mac OS X operating systems. In addition to Java, PLASMID uses code written using the C++ programming language. C++ is needed for computationally intensive tasks that require greater speed and efficiency. The use of two different programming languages is transparent to the user.

PLASMID provides an integrated environment for designing an optimal classification microarray. As such, PLASMID v0.91 includes the following services:

1. Loading and management of different kinds of input data, including plasmid sequence data, hybridization data, virtual hybridization data, and probe sequences. Data may be in tab-delimited or comma-delimited text format or in Microsoft Excel spreadsheet format.

2. Different methods for processing hybridization data. The tool provides several data preprocessing methods, including normalization and noise filtering. It also provides hierarchical, non-hierarchical, and model-based methods for clustering samples; two different statistical tests for ranking probes; use of K-means clustering for reduction of probe redundancy; and stepwise discriminant analysis with assignment of weights to probes.

3. Design of mixed arrays using existing hybridization data or virtual hybridization data. An optimal set of probes is identified, and weights associated with each probe are stored for analysis of experimental results.

4. Construction of virtual microarrays to obtain virtual hybridization data using genomes from the National Center for Biotechnology Information (NCBI) database. Genomes for probes can be chosen by accession number or by gene sequence.

5. Visualization of microarray data and data processing results, including dendrograms, heat maps, and scatter plots. Plots can be saved in different image formats.

6. Automatic probe design after the user has specified the parameters. A step-by-step wizard guides the user through the various steps.

Experimental data obtained from microarrays designed using PLASMID can be used as input data and analyzed using the weighted classification function obtained in 3.

## Results and Discussion

In this section we present results obtained using PLASMID to analyze a mixed-plasmid microarray data set [4] and a simulated mixed-genome microarray data set [1]. We also present results for publically-available leukemia expression array data [18]. For this latter data set, clusters (i.e., types of leukemia) are pre-assigned so only probe ranking, reduction of probe redundancy, and stepwise discriminant analysis (SDA) are used to determine the optimal probe set. PLASMID's performance in probe selection is evaluated using the leave-one-out (LOO) approach for which one sample is excluded and the remaining samples are used to obtain the discriminant functions. Each sample is, in turn, excluded and a corresponding set of discriminant functions is used to classify it. The prediction accuracy, the percentage of times the withheld samples are correctly classified, is used as the performance metric.

### Mixed-plasmid microarray data

A mixed-plasmid microarray has been used to compare the genetic composition of plasmids [4]. The microarray consists of 576 probes composed of randomly selected fragments of plasmid DNA, and the data were obtained from hybridization experiments with 43 plasmids. The data are composed of hybridization signal intensities for each microarray probe [see Additional file 1].

**Figure 2:**
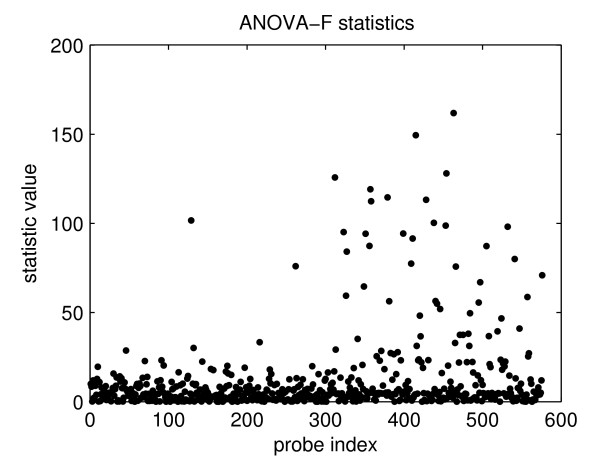
**Scatter plot of ANOVA-*F *test statistics for the mixed-plasmid microarray probes**. The scatter plot shows that the majority of the probes have statistical values close to zero and, thus, that ANOVA-*F *test statistics can be used to distinguish between informative (*F *> 0) and uninformative (*F *≈ 0) probes.

First we used the Ward's minimim variance hierarchical clustering algorithm to create a dendrogram. To test the two-class problem, we divided the dendrogram into two clusters. One cluster consisted of 15 plasmids which, with one exception (the *peSSuTet *plasmid), have the *bla*_CMY-2 _antibiotic resistance gene; the other cluster consisted of 28 plasmids. We then used the probe ranking function, choosing the ANOVA-*F *test statistic, and generated a scatter plot (Fig. 2). The scatter plot shows that the majority of the probes have statistical values close to zero and, thus, that ANOVA-*F *test statistics can be used to distinguish between informative (*F *> 0) and uninformative (*F *≈ 0) probes. This result also serves to highlight the need for optimization algorithms, as the majority of probes provide limited discrimination.

For the two-cluster case, we chose 1, 20, and 200 top-ranked probes for comparison. Using reduction of probe redundancy and SDA, we found that a single probe (5-E3, a transposase gene associated with the *bla*_CMY-2 _element [19]) correctly classified all but two of the plasmids [4]. Interestingly, in the original study one of these two plasmids (*pe1171sT*) was classified with plasmids that harbor the *bla*_CMY-2 _gene even though it does not carry this gene. Analysis with PLASMID separated *pe1171sT *from the *bla*_CMY -2 _plasmids. In addition, a different plasmid (*pe7594T*) that harbors the *bla*_CMY-2 _gene was classified with other *bla*_CMY-2 _positive plasmids. Thus, analysis using PLASMID more accurately reflects the phenotypic properties of the plasmids included in the study. The one exception was the *peSSuT *plasmid that was consistently classified with *bla*_CMY-2 _plasmids while not harboring this gene [8].

Next we divided the original dendrogram into five plasmid clusters and ranked probes as before. As expected, the number of probe clusters *κ *specified for the reduction of probe redundancy affects the prediction accuracy (Table 1). Small values of *κ *certainly reduce redundancy, but they also reduce specificity. The optimal set of probes is identified using SDA with the LOO method to determine the highest prediction accuracy. In this case, the smallest number of probes from the top-most ranked probes with the highest prediction accuracy is 10. Thus, PLASMID analysis reduced the original data set of 576 probes to 10 probes that are needed to accurately classify plasmids into one of five groups. Non-hierarchical clustering followed by probe ranking, probe reduction, and SDA gave similar results (data not shown).

**Table 1:** Classification accuracy of mixed-plasmid data using hierachical clustering with five sample (plasmid) clusters. PA is the prediction accuracy.

	Number of clusters of probes, *κ*											
	
	2		5		10		20		30		40	
	
Number of top-ranked probes	PA (%)	No. of probes	PA (%)	No. of probes	PA (%)	No. of probes	PA (%)	No. of probes	PA (%)	No. of probes	PA (%)	No. of probes
100	72.09	2	72.09	5	72.09	10	69.77	19	69.77	29	69.77	32
150	86.05	2	93.02	5	95.35	10	95.35	20	95.35	29	95.35	36
200	74.42	2	90.70	5	93.02	10	93.02	20	93.02	30	95.35	35
250	76.74	2	95.35	5	95.35	10	95.35	20	95.35	30	90.70	34
300	46.51	2	88.37	5	93.02	10	93.02	20	93.02	30	90.70	35
350	76.74	2	93.02	5	93.02	10	95.35	20	95.35	30	95.35	33
400	69.77	2	93.02	5	90.70	10	93.02	20	90.70	30	93.02	35

In addition to hierarchical and non-hiearchical clustering methods, we can obtain classification results using our model-based method, which is based on a genetic algorithm. The genetic algorithm predicted that the most likely number of plasmid clusters is five (Table 2). Comparison of Tables 1 and 2 shows that prediction accuracies depend on the initial clustering method used. For this case, the prediction accuracies for the model-based clustering method are larger for a given number of probe clusters than those obtained via the hierarchical method. Furthermore, the variance in prediction accuracies is lower as a function of the number of top-ranked probes when clusters are initially assigned using the model-based method. For other data sets, however, another clustering model might give the best results.

**Table 2:** Classification accuracy of mixed-plasmid data with model-based clustering. PA is the prediction accuracy.

	Number of clusters of probes, *κ*											
	
	2		5		10		20		30		40	
	
Number of top-ranked probes	PA (%)	No. of probes	PA (%)	No. of probes	PA (%)	No. of probes	PA (%)	No. of probes	PA (%)	No. of probes	PA (%)	No. of probes
100	83.72	2	95.35	5	95.35	10	95.35	19	95.35	36	95.35	33
150	53.49	2	90.70	5	93.02	10	93.02	20	93.02	36	93.02	28
200	79.07	2	93.02	5	93.02	10	93.02	20	93.02	36	93.02	35
250	76.74	2	95.35	5	95.35	10	95.35	20	95.35	35	93.02	32
300	69.77	2	93.02	5	93.02	10	95.35	20	95.35	34	95.35	35
350	67.44	2	93.02	5	93.02	10	93.02	20	93.02	35	93.02	35
400	69.77	2	93.02	5	93.02	10	93.02	20	93.02	37	95.35	35

Based on the sample-to-feature ratio (SFR), at least 9 probes (features) are required for classifying 43 plasmids (samples). Tables 1 and 2 show several choices for 10 probes with equivalent performance. When additional information is available, it should be used to assist with the choice of a final set.

### Virtual Streptococcus mixed-genome microarray data

A virtual *Streptococcus *mixed-genome microarray was constructed by Wan *et al*. [1]. To create the equally-represented, 4000-probe virtual array, 800 gene segments each 600-bp long were randomly selected from genomes of fifteen strains of five bacterial species–that is, each species was represented by 800 different probes. Virtual hybridization was accomplished using BLAST scores as proxies for array probe intensities [see Additional file 2], and PLASMID was used to analyze the data. In the initial analysis one bacterial species was excluded from the study because it was represented by only a single strain (*S. mutans *UA159). Because we knew a *priori *that the samples belonged to four different species, the goal was to find an optimal set of probes to classify these four. ANOVA-*F *tests were used to rank the 4000 probes, and LOO analysis was performed on different numbers of the highest ranked probes. In fact, we found the LOO prediction accuracy to be 100% for differentiating the four different species using only the single top-ranked probe. On examination we found that the hybridization values (BLAST scores) for this probe for strains from different groups were well separated (*i.e*., different from each other), while the hybridization values for strains from the same group were very similar. While it appears that successful classification can be achieved with a single probe when classification relies on differences in hybridization signal, given inherent sources of variation in microarray hybridization, it would be prudent to include additional probes to increase classification confidence for empirical data. For example, the minimum recommended probe set in this case would be 3 according to the SFR.

**Figure 3:**
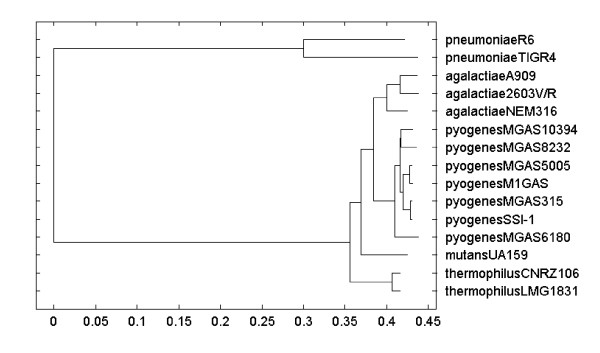
**Dendrogram for *Streptococcus *MGM data**. The dendrogram constructed using the neighbor joining method shows a clear distinction between the two *S. pneumoniae *samples and the remaining bacteria.

In the second analysis, our model-based clustering method identified two clusters, one with the two *S. pneumoniae *strains and the other with the remaining 13 strains. After probe ranking, reduction of probe redundancy, and SDA, a single probe could be used to differentiate these two groups. We also used non-hierarchical clustering of the samples followed by probe ranking, probe reduction, and SDA. When the number of clusters was chosen to be *k *= 2, the result was identical to the result obtained using our model-based cluster method. When the number of clusters was chosen to be *k *= 3 or *k *= 4, the two *S. pneumoniae *genomes were placed into different groups. A dendrogram constructed using the neighbor joining method shows a clear distinction between the two *S. pneumoniae *samples and the remaining bacteria (Fig. 3). When these two samples are excluded, PLASMID groups the remaining thirteen samples correctly into four species clusters. The results shown in Table 3 are obtained using non-hierarchical clustering, probe ranking, probe reduction, and SDA. As this table illustrates, only 2 probes are needed to obtain 100% prediction accuracy by species. These 2 probes are from the genomes of *S. pneumoniae *TIGR4 and either *S. pyogenes *M1 GAS or *S. pyogenes *MGAS5005. Based on the SFR rule of thumb, at least 3 probes are needed. Several choices exist that suffice for this condition.

**Table 3:** Classification accuracy using mixed-genome array data with non-hierarchical clustering for four sample (bacterial species) clusters. PA is the prediction accuracy.

	Number of clusters of probes, *κ*											
	
	2		5		10		20		30		40	
	
Number of top-ranked probes	PA (%)	No. of probes	PA (%)	No. of probes	PA (%)	No. of probes	PA (%)	No. of probes	PA (%)	No. of probes	PA (%)	No. of probes
50	100	2	100	5	100	7	100	7	100	1	75	1
100	100	2	100	5	100	7	100	7	100	7	100	1

For virtual microarrays, BLAST scores are used to obtain hybridization intensities, and the accuracy of the scores will affect the choice of an optimal probe set. While error could be modeled from real data, the best measure of reliability will be obtained using actual hybridization experiments.

### Public ALL/AML leukemia data

The ALL/AML leukemia data set, obtained from expression arrays, has been widely used in the literature. It consists of two classes of leukemia, acute lymphoblastic leukemia (ALL) and acute myeloblastic leukemia (AML), and there are 72 samples (47 ALL and 25 AML) and 7129 probes. Table 4 shows prediction accuracy results after probe ranking, probe redundancy reduction, and SDA have been performed. When the top 50 probes were selected, the highest accuracy was achieved when probes were clustered into 10 groups. A set of 10 probes was identifed with a prediction accuracy of 97.22%. Using additional probes does not lead to improvement. According to the SFR rule of thumb, at least 20 probes should be used in the actual microarray design; several choices of 20 probes exist and all produce robust prediction results (Table 4).

**Table 4:** Classification accuracy using ALL/AML leukemia data. PA is the prediction accuracy.

	Number of clusters of probes, *κ*											
	
	2		5		10		20		30		40	
	
Number of top-ranked probes	PA (%)	No. of probes	PA (%)	No. of probes	PA (%)	No. of probes	PA (%)	No. of probes	PA (%)	No. of probes	PA (%)	No. of probes
50	94.44	2	94.44	5	97.22	10	97.22	20	97.22	30	97.22	39
100	88.89	2	95.83	5	94.44	10	97.22	20	97.22	30	97.22	40
150	83.33	2	95.83	5	97.22	10	97.22	20	97.22	30	97.22	39
200	79.17	2	80.56	5	97.22	10	97.22	20	97.22	30	97.22	39
250	79.17	2	79.17	5	97.22	10	97.22	20	97.22	30	97.22	39

## Conclusion

In this paper we describe a new software tool, PLASMID, for selecting an optimal set of probes for the design of a classification microarray. The tool provides the user with several clustering methods, a probe ranking method, probe redundancy reduction, and probe selection using stepwise discriminant analysis. Images can be saved in several different formats, and weights generated using SDA can be stored for use in analysis of experimental data. In addition, PLASMID can be used to construct virtual microarrays with genomes from public databases; these can then be used to determine an optimal probe set for use in actual microarray experiments. The software package has been applied to data from a mixed-plasmid microarray, a virtual mixed-genome microarray, and an expression microarray. Robust results have been obtained for all three sets of data.

Although many methods are available for determining a set of probes for a given microarray data set, these methods require the classification information to be known in advance. PLASMID was designed to be used prior to implementation of a microarray when no such information is available, although the program can also be used when clusters are known a *priori*.

PLASMID can be obtained by following the link from .

## Availability and requirements

• Project name: PLASMID

• Project home page: 

• Operating system: Windows but to be ported to Linux and Mac OS X

• Programming languages: Java and C++ (with gcc compiler)

• Other requirements: Java Runtime Environment

• License: Free to academic and nonprofit organizations

## Authors' contributions

DM and SLB developed PLASMID, DM was responsible for programming PLASMID, and DRC provided the necessary microbiology expertise. All authors have read and approved the final manuscript.

## Supplementary Material

Additional file 1 **MPM**. This file contains the information for the mixed-plasmid microarray in the form of a 44-row × 577-column matrix. The first row gives the 576 probe names, and the first column gives the 43 plasmid names. The remaining rows and columns contain the hybridization intensity data. Click here for file

Additional file 2 **MGM**. This file contains the information for the mixed-genome virtual microarray in the form of a 15-row × 4001-column matrix where the first column gives the 15 names of the streptococcus strains. The probes were not given specific names. All remaining columns contain the virtual hybridization intensity data. Click here for file
